# E-learning for chest x-ray interpretation improves medical student skills and confidence levels

**DOI:** 10.1186/s12909-018-1364-2

**Published:** 2018-11-12

**Authors:** S. Wentzell, L. Moran, J. Dobranowski, A. Levinson, A. Hannigan, C. P. Dunne, D. McGrath

**Affiliations:** 1 Graduate Entry Medical School, University of Limerick, Limerick, Ireland; 2 Faculty of Health Sciences, McMaster University, Hamilton, ON Canada

**Keywords:** E-learning, Radiology, Undergraduate curriculum, Online resource, Medical curriculum, Medical students, X-ray interpretation, Chest X-ray, Modules

## Abstract

**Background:**

Radiology is an important aspect of medicine to which medical students often do not receive sufficient exposure. The aim of this project was to determine whether the integration of an innovative e-learning module on chest x-ray interpretation of the heart would enhance the radiological interpretive skills, and improve the confidence, of first year graduate entry medical students.

**Methods:**

All first-year graduate entry (all students had a prior university degree) medical students at the University of Limerick (*n* = 152) during academic year 2015–16 were invited to participate in this study. An assessment instrument was developed which consisted of 5 radiological cases to be interpreted over a designated and supervised 15-min time period. Students underwent a pre-, mid- and post-intervention assessment of their radiology interpretative skills. An online e-module was provided following the pre-test and additional practice cases were provided following the mid-intervention test. Assessment scores and confidence levels were compared pre-, mid- and post-intervention.

**Results:**

The overall performance (out of a total score of 25) for the 87 students who completed all three assessments increased from 13.2 (SD 3.36) pre-intervention to 14.3 (SD 2.97) mid-intervention to 15.8 (SD 3.40) post-intervention. This change over time was statistically significant (*p* < 0.001) with a medium effect size (eta-squared = 0.35). Increases from pre- to post-intervention were observed in each of the five areas assessed, although performance remained poor in diagnosis post-intervention. Of the 118 students who provided feedback after the intervention, 102 (86.4%) stated that they would recommend the resource to a colleague to improve their interpretative skills.

**Conclusions:**

This study suggests that early exposure to e-learning radiology modules is beneficial in undergraduate medical school curricula. Further studies are encouraged to assess how long the improvement may last before attrition.

**Electronic supplementary material:**

The online version of this article (10.1186/s12909-018-1364-2) contains supplementary material, which is available to authorized users.

## Background

Radiology is a medical specialty that, per previous studies, very few medical students receive adequate exposure to in their undergraduate curriculum, particularly in the pre-clinical years [1]. Recent data suggest that 5% of total teaching time is dedicated in medical schools to the subject of radiology [2] with the majority of teaching taking place in the later years and only 20% of schools in Europe reporting having radiology teaching in the first year of their programmes [3]. Literature has also shown that students lack confidence when expected to interpret radiological images [4]. This suggests that undergraduate radiology teaching does not adequately meet the needs of students and potentially leaves them ill-prepared for clinical rotations [2]. Researchers have tried to seek ways to improve this deficit through the development of specific objectives [5] and a range of online resources [6, 7]. However, students continue to criticize the lack of radiology teaching in the early undergraduate years [1]. Furthermore, there appears to be limited emphasis and research on the importance of diagnostic imaging interpretative skills in the early stages of the undergraduate medical curriculum.

To date, radiology teaching has been delivered via a range of platforms – lectures, small group case-based discussion sessions, problem-based learning and more recently through online/e-learning resources [8]. It is well documented that online learning provides an active learning environment particularly when delivered in a case-based or problem-based learning format [9]. Radiology, by nature of its imaging techniques, is a discipline that lends itself well to this method of learning [10]. However, interactive feedback is critical to the success of this method of learning and to the mastery of radiology skills [8].

The aim of this project was to explore the level of radiological knowledge of first year graduate entry medical students and to determine whether the integration of an interactive e-learning module encompassing the interpretation of the heart on chest radiography early in the programme would enhance student interpretation skills and confidence levels.

## Methods

### Setting

The University of Limerick (UL) offers a four-year graduate entry medical degree programme leading to the award of Bachelor of Medicine, Bachelor of Surgery (BM BS). Students therefore enter the programme having completed a prior university degree. In the case of the programme at UL to be eligible for entry candidates must hold a minimum 2.1 (second class honours, grade one) result in their first National Framework Qualification (NFQ) Level 8 major Award Honours Bachelor Degree. Students from any discipline may apply (i.e. there is no pre-requisite for science over non-science background).

Upon completion of the course students are required to achieve the same competencies as in longer, traditional medical degree programmes. Hence the programme is more intensive.The first two (“pre-clinical”) years of the programme utilize a systems-based approach to the basic sciences, which centres on problem-based learning (PBL). Years 3 and 4 of the programme provide students with an intensive clinical apprenticeship, encompassing the major clinical disciplines and General Practice.

The Basic Sciences and Clinical Sciences are integrated throughout all 4 years with a strong emphasis on self-directed learning. The curriculum for years 1 and 2 (referred to as the ‘Year 1 & 2’ curriculum) provides students with a thorough grounding in the basic sciences relevant to medicine through the study of 66 carefully designed, customised PBL cases. In keeping with the integrated nature of the course, the PBL tutorials in Year 1 & 2 are supplemented by weekly clinical and anatomical-skills teaching sessions covering topics directly related to the PBL case for that week.

Radiology teaching in the early years of the programme has traditionally been included in the anatomy programme and has focused on learning radiological anatomy and recognising normal images relevant to the PBL case of the week. This format is complemented by lectures regarding the underlying principles and concepts of radiology with minimal attention to the interpretation of images. In the later years, greater emphasis is placed on the interpretation of abnormal images. Due to the PBL nature of the programme in which there is exposure to diagnostic images a not unrealistic demand for some teaching in the area of imaging interpretation has therefore arisen.

### Subjects

Participants of this study were recruited from the Graduate Entry Medical School (GEMS) at the University of Limerick in Ireland. All students in Year 1 (first year) in the academic year 2015–16 were invited to participate (*n* = 152). The student median age was 24 years (range 21 to 42 years) with the majority (77%) having a primary university degree in a science-related subject. Two thirds of the students are from Europe with the majority of the others from Canada.

### E-learning module

An online web-based intervention, developed by McMaster University was employed. The module was aligned with the Year 1 Cardiology & Respiratory (“Life Support”) learning unit which incorporates cardiothoracic anatomy and pathology and was introduced after completion of formal sessions on the anatomy of the heart, lungs and mediastinum. The E-learning module consisted of two parts:

The first part of the e-learning module incorporated basic radiological physics (how an x-ray is formed, types of x-rays, range of x-ray interfaces) to help students understand why a chest x-ray image appears as it does. This aspect of the module focused on the radiological anatomy of the heart as visualised on posterior-anterior (PA) and lateral chest x-ray and consisted of a step-by-step approach to interpreting the heart on chest x-ray. This approach described a systematic and methodical technique for x-ray interpretation integrated in case scenarios [11]. This part of the module specifically covered the definition and interpretation of grayscale appearance (ie level of image intensity on chest x-ray in varying shades of grade with black as the weakest and white as the strongest intensity [11]) in addition to heart size, shape and position and was run in parallel with a problem based the module provided a resource on interpreting Congestive Heart Failure (CHF) on a chest x-ray.

The second part of the e-learning module (focussing on interpretation and diagnosis) consisted of 13 radiology cases comprising images of common chest pathology, each of which had a cardiac focus. When working through these cases, students were provided with step by step feedback and answers, similar to the first part of the e-module. This provided a blocked practice intervention for students focusing solely on cardiac interpretation. Cases used in this part of the intervention included CHF, pericardial calcification, prosthetic valves, cardiomegaly and normal chest x-rays.

The module was delivered in an interactive power point format and integrated into the Medical School’s student virtual learning environment. All first-year students were given access to both parts of the module via an online web-based portal accessed with the students’ identification numbers and passwords. Students were therefore able to access via their own personal devices and home computers. Students had access to the first part of the module for 1 month prior to be given access to the second part of the module. The students therefore had access to the e-learning and interpretative/diagnostic module for a total period of 3 months. A time of 1 h was anticipated to complete the module. Immediate feedback was the form of an explanation of the correct response was given to students as they proceeded through the on-line module.

### Assessment of E-learning module

An assessment instrument was developed which consisted of 5 radiological cases to be interpreted over a 15-min time period. Each case demonstrated either a normal or abnormal chest x-ray. These cases were selected as commonly encountered “not to miss” conditions seen on imaging during clinical training and matched the instructional content.

As this was an introduction to a new resource, being used for formative purposes and, in an effort to reduce the burden on students, it was decided to limit the assessment to a select number of cases encompassing important “not to miss” diagnoses. Instructions were provided to the learners/test-taker.

**Fig. 1:**
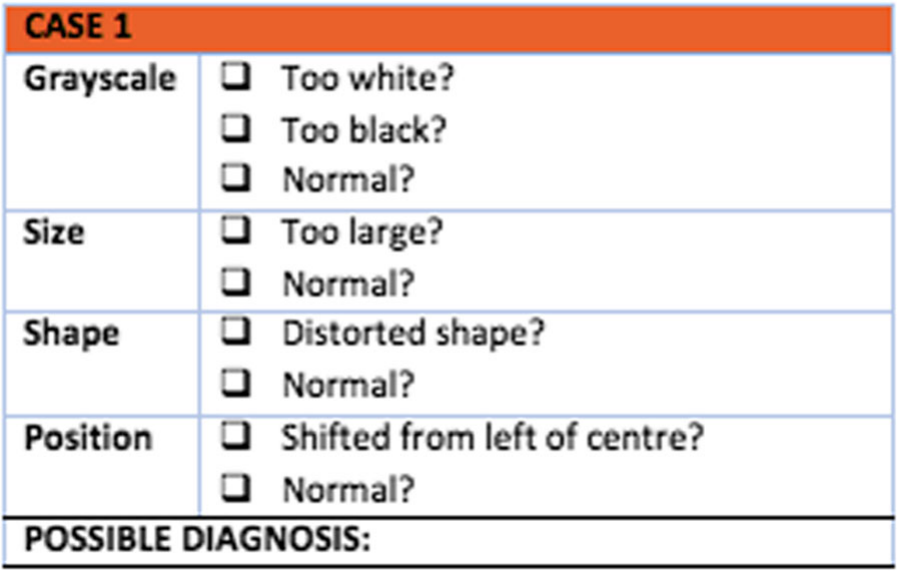
Assessment instrument. Scoring of chest x-ray case based on overall grayscale appearance of the image, size, shape and position of the heart and diagnosis

Students were not informed of the number of normal/abnormal cases. Students were required to describe each chest x-ray based on overall grayscale appearance of the image, size, shape and position of the heart and diagnosis (see -Fig. 1). The criteria chosen in this case were informed by the “Descriptive Phase” previously reported by Dobranowski et al. [12]. The instrument was reviewed by a Radiologist to ensure ease of use and correct understanding.

One mark was allocated for each correct answer giving a maximum total score of 5 marks for each case and 25 in total (see Additional file 1: Appendix 1). Students were required to complete the assessment at a specified time under supervision.

**Fig. 2:**
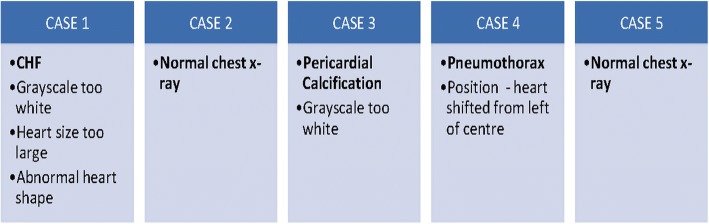
Sequence of cases and associated features. This figure depicts the sequence of cases (Case 1,2,3,4,5) provided to the students

The 5 cases were sequenced in a manner, which would stimulate an interpretative process and ensure all marking criteria were covered (see Fig. 2). These specific case images were not incorporated into the e-learning module.

Each case was presented as an image on a projector screen in a lecture hall and under faculty supervision with all participating students (*n* = 152) completing the assessment in hard copy format at the same time. At no point in time did students have access to the assessment cases outside of the supervised assessment setting. The assessment was for formative purposes and did not contribute to students’ overall grades. Students were not given solutions or any feedback on their assessment at baseline or mid intervention. This allowed the same 5 cases to be used for each assessment, encouraging the use of interpretative skills rather than the memorization of answers. A single assessor marked all students.

### Student evaluation of E-learning module

A questionnaire (see Additional file 2: Appendix 2) was made available for participants to provide feedback regarding their confidence in interpreting chest x-ray images with particular focus on interpretation of the heart. The questionnaire was developed by an expert academic radiologist, Instructional designer/med educational researcher and a member of the target (medical student) audience and was mapped to the key clinical tasks.

The questionnaire Confidence levels were recorded on a Likert type scale of 1–5 with 1 being not at all confident and 5 being extremely confident.

### Study timeline

The questionnaire with assessment instrument integrated was delivered in hard copy format prior to the e-learning module being made available in the University of Limerick curriculum (pre-intervention assessment). The pre-intervention assessment allowed for baseline scores, reflecting the level of skill/knowledge at commencement of the intervention, to be recorded. All Year 1 students completed it at a mandatory time and location under supervision.

The first part of the e-learning module was then released to all Year 1 students. Students had full access to the module for the duration of the study (i.e. total of 3 months) and were able to revisit the module as often as they felt necessary.

The integrated questionnaire (again in hard copy format), was delivered 1 month after the pre-intervention assessment, at a specified time and location and under faculty supervision (mid-intervention assessment). The same five cases were used again.

Full access was then given to the second part of the e-learning module i.e. an additional resource of practice cases. This ‘practice cases’ component provided students with 13 additional cases to solidify their interpretative abilities. Students had access to this part of the e-learning module for the remainder of the study.

Finally, the integrated questionnaire was delivered 2 months later (hard copy format), at a specified time and location and under faculty supervision (post-intervention assessment). The same five test cases as before formed the basis of the assessment. Additionally, students were asked whether they found the e-learning modules helpful and if they would like more radiological learning delivered. They were also encouraged to give comments or suggestions based on their experience with the chest x-ray interpretation of the heart e-learning module.

### Ethics

The University of Limerick’s, Faculty of Education & Health Sciences Research Ethics Committee approved the approach whereby completion of the integrated questionnaire indicated consent (Approval No 2014_12_07_EHS).

### Data analysis

The assessments were manually scored by the first author and all data entered into Microsoft Excel. The distribution of assessment scores and confidence ratings were tested for normality and summarised using mean (standard deviation) for normal distributions. A repeated-measures analysis of variance (ANOVA) was used to test for changes in assessment scores and confidence ratings over time (pre-, mid- and post-intervention assessment). The assumptions underlying repeated-measures ANOVA i.e. multivariate normal distribution and sphericity were checked using probability plots and Mauchly’s test. Eta squared was used to measure effect size for change in total assessment score over time. Paired t tests were carried out on differences in assessment scores between pre- and mid-intervention, pre- and post-intervention and mid- and post-intervention, with a Bonferroni correction used to adjust for multiple testing. Spearman’s rank correlation coefficient (r_s_) was used to measure the strength of the association between rating of confidence and assessment score in each assessed area. SPSS Statistical Software for Windows Version 22 was used for the statistical analysis. Free text comments from students were independently coded into themes by three of the authors and a consensus reached on the themes emerging.

## Results

### Response rates

Of the 152 students in Year 1, 143 (94%) completed the pre-intervention assessment. Of these 143 students, 111 (78%) completed the mid-intervention assessment, 103 (72%) completed the post-intervention assessment and 87 (61%) completed all three assessments. The fixed times for the three assessments resulted in some students not being available at all time points but there was no difference in mean pre-intervention assessment scores between those who participated in the post-intervention assessment and those who did not participate (mean of 13.0 vs. 13.1, *p* = 0.87).

### Assessment performance

The overall performance (out of a total score of 25) for the 87 students who completed all three assessments increased from 13.2 (SD 3.36) pre-intervention to 14.3 (SD 2.97) mid-intervention to 15.8 (SD 3.40) post-intervention. This change over time was statistically significant (*p* < 0.001) with a medium effect size (eta-squared = 0.35). The changes in overall performance from pre- to post-intervention and from mid- to post-intervention were statistically significant (mean difference of 2.4, 95% confidence interval 1.6 to 3.1, p < 0.001 and mean difference of 1.5, 95% confidence interval 0.7 to 2.2, *p* < 0.001 respectively). The difference between pre- and mid-intervention was not statistically significant (*p* = 0.06).

Increases from pre- to post-intervention were observed in each of the five areas assessed (Table 1) although performance was still poor in diagnosis post-intervention.

**Table 1:** Performance in each area assessed (out of 5) pre-, mid- and post-intervention (*n* = 87 students)

	Pre-intervention mean assessment score (SD)	Mid-intervention mean assessment score (SD)	Post- intervention mean assessment score (SD)	*p*-value^1^
Grayscale	3.1 (1.02)	3.1 (1.03)	3.4 (0.90)	0.025
Size (PA^a^ view)	3.3 (1.42)	3.8 (1.16)	4.0 (1.13)	< 0.001
Shape	2.6 (1.14)	2.9 (0.85)	3.2 (1.05)	< 0.001
Position	3.6 (1.17)	3.8 (0.97)	4.0 (1.08)	0.049
Diagnosis	0.6 (0.81)	0.6 (0.84)	1.2 (1.27)	< 0.001

^1^From repeated measures ANOVA

^a^Posteroanterior

### Student evaluation

Students were also asked to rate their confidence with regard to ability to complete the tasks in Table 2 on a scale of 1 (not at all confident) to 5 (extremely confident). The mean confidence ratings pre-, mid- and post- intervention are given in Table 2. Confidence levels for all tasks increased post-intervention, although post-intervention confidence levels were still relatively low with most below 3 on a 5 point scale.

**Table 2:** Confidence ratings (out of 5) pre-, mid- and post-intervention (*n* = 87 students)

	Pre-intervention mean confidence rating (SD)	Mid-intervention mean confidence rating (SD)	Post intervention mean confidence rating (SD)	*p*-value^1^
Determining if the CXR is normal or abnormal	1.8 (0.90)	2.3 (0.98)	2.5 (1.00)	< 0.001
Determining if the heart is too white, too black or of normal grayscale	1.8 (0.94)	2.3 (0.97)	2.6 (1.05)	< 0.001
Determining if the heart is too large or of normal size	1.8 (0.93)	2.4 (0.97)	3.0 (1.10)	< 0.001
Determining if the heart is of normal or distorted (abnormal) shape.	1.7 (0.83)	2.2 (0.92)	2.7 (1.09)	< 0.001
Determining if the heart is in a normal anatomical position or if it has shifted	2.1 (0.99)	2.6 (1.03)	3.0 (1.13)	< 0.001
Giving a differential diagnosis based on the image findings	1.3 (0.57)	1.4 (0.66)	1.7 (0.81)	< 0.001
Overall interpretation of the heart on a CXR	1.5 (0.64)	1.8 (0.68)	2.1 (0.94)	< 0.001

^1^From repeated measures ANOVA testing the hypothesis that there is no difference over the three time points

There was a weak, positive correlation between rating of confidence and assessment score post-intervention for grayscale, size, shape, and position (r_s_ ≤ 0.30) with a stronger correlation between rating of confidence in diagnosis and assessment score for diagnosis (r_s_ = 0.44, *p* < 0.001).

One hundred eighteen students provided feedback after the intervention with 102 (86.4%) stating that they would recommend the resource to a colleague to improve their interpretative skills. 114 (96.6%) indicated that more tutorials on chest x-ray interpretation would benefit their learning.

Forty-one students provided free text comments on anything additional that could be incorporated into the tutorials to benefit their learning. The themes emerging from these comments related to positive feedback on how useful the resource was and how it motivated further learning; the need to assess it and make it mandatory and the need for further resources and integration with existing teaching and the curriculum (Table 3). Only one negative comment was given on how the resource was not necessary at this stage of the curriculum.

**Table 3:** Feedback from students

Positive feedback	Constructive feedback	Negative feedback
*General Feedback* • Helped to understand X-rays • It’s quite good and goes into a lot of detail without going overboard • It was a really helpful resource • Clear and concise • Good job at breaking down the x-rays • I now understand what I am looking for... • The powerpoints are very good with step by step guidance. Easy to follow and repetitive so you understand it more • *(Since we don’t have any other formal imaging interpretation)*, this was very enjoyable and beneficial • It’s a useful skill for clinical practice so this helped develop my learning • It was very useful in increasing knowledge and recognition from baseline *Expression of interest in further modules* • I would like to learn more • More modules please • Other tutorial and images would be beneficial (fractures, brain, lungs)	*Mandatory* • Make it mandatory • The fact that we are not examined on this makes it difficult to prioritize it • The school should make it mandatory so everyone reviews it and benefits *Aligning with Curriculum* • Need to incorporate into clinical skills, we don’t have the time to do it on our own time • It would be nice to have someone to ask questions too • This tutorial approach should be supplemented with a lecture	• Unnecessary at this stage

## Discussion

The aim of this project was to determine whether the integration of an innovative e-learning module on chest x-ray interpretation of the heart would enhance the radiological interpretive skills, and improve the confidence, of first year graduate entry medical students.

The findings demonstrated a modest improvement in basic chest x-ray interpretation skills and confidence levels amongst first year graduate entry medical school students following the introduction of an e-learning module. While the ability for students to make a diagnosis did improve performance on this assessment item was poor. One explanation for this is that the study was conducted over a short time frame with Year 1 medical students who had minimal, or no, prior exposure to medicine and radiology. As students gain further medical knowledge, one would assume that both skills (including diagnostic skills) and confidence levels at x-ray interpretation would increase more dramatically with additional formal e-learning modules delivered throughout subsequent years of the programme. These data are consistent with a previous study by Wong et al. in which higher assessment scores in response to adaptive (e-learning) diagnostic imagine modules were driven by higher assessment scores in senior as opposed to junior students [13].

Student perceptions of the e-learning module were generally positive with the majority indicating that they would recommend it to their peers and some students requesting more radiology modules in the first year of the programme. This differs from the study by Nyhsen et al. who reported a lack of satisfaction with e-learning radiology training modules by medical students in their clinical training years [1]. Nyhsen et al. however, acknowledged that the students in their study had very little on-line access to updated, effective e-learning modules. The additional improved performance demonstrated after exposure to the ‘practice cases’ in our study (which was not available to the students in Nyhsen et al’s study) further supports the theory that the application of knowledge and skills is critical to learning. This theory is also supported by Maleck et al. [6] who conducted a similar study assessing medical students’ radiology interpretation skills following the integration of case studies; this showed that computer-based didactic teaching is not alone sufficient.

Comparing the pre-intervention versus the post-intervention data in this study, there is a benefit of teaching x-ray interpretation through an e-learning module. It is difficult to determine whether the resource alone caused the increase in overall score and confidence or whether it stimulated students to research and practice on their own time outside of the e-learning module. Additionally, as this study only focused on one type of intervention (an e-learning module) and not other methods of delivery, the improved performance could also represent an increase in general exposure to existing radiology teaching (as previously described in the Methods section) as students progressed through the academic year. Of interest is the fact that there was an increase in overall score following the release of the practice cases. This suggests that it is not enough to simply retain material from a single instructional module but that there is a need for students to be provided with opportunities to apply new knowledge and skills. This supports the problem-based learning pedagogy where students are encouraged to be self-directed and motivated to acquire knowledge and skills relevant to their educational needs and future careers [14]. It is proposed therefore that medical programs be encouraged to promote radiology teaching that is integrated as a longitudinal thread in a spiral curriculum and which has clear emphasis on interpretive skills relevant to future clinical practice.

While understanding radiology concepts (role, indications and appropriate ordering) is important in providing a knowledge basis for the discipline we would argue that there is also a need for greater emphasis on the interpretation of images early in undergraduate medical curricula particularly as more medical degree programmes adapt PBL or variants thereof.

Free –text comments from this study have also illustrated a desire amongst junior medical students to obtain more exposure to the specialty of radiology, in particular interpretative skills, early on in their medical education. This situation whereby “pre-clinical” students are requesting instruction on imaging interpretation will need to be addressed as more medical degree programmes adapt PBL or variants thereof, as a method for facilitating learning. While the students in this study admitted difficulty in finding time to complete the e-learning modules, free text comments also suggest that there is a desire amongst students for this type of e-learning module to be made mandatory and be assessed. Given the vast amount of medical knowledge students must retain for the purpose of undergraduate examinations, teaching that is not assessed is often not made a priority [15–17].

Properly interpreting an x-ray can ultimately significantly impact the management and treatment of a patient [12]. Despite the critical nature of radiological knowledge and skill however, most often radiology teaching is incorporated into the medical curriculum as an adjunct [8]. With limited and poorly integrated radiological teaching in medical curricula, it is not surprising therefore that students are uncomfortable when presented with an x-ray [4]. This may provide one of many reasons why there has been a decrease in residency applications for the specialty of radiology despite the increasing number of training places being made available in the United States [18]. While the authors acknowledge that imaging interpretation requires expertise, when learning in the context of real patient clinical problems such as that found in programmes that incorporate PBL pedagogical methods we would argue that students should have the opportunity to familarise themselves with and become more confident in the skill of image interpretation from early on in their training. By progressing through both an undergraduate and post graduate curriculum students then have the opportunity to move through the Eraut’s 5 stages of skills acquisition [19].

There is a need therefore for early, integrated, radiology teaching in medical curricula with a view to better preparing students for the real-life clinical environment.

### Limitations

The researchers acknowledge that the cohort analyzed comprised first year medical students with minimal experience in radiology and hence the comments they provide may not be representative of other years. A more detailed qualitative study of the perspectives of both junior and senior medical students with respect to radiology teaching is therefore necessary. One might also argue that the items assessed were too technical and too advanced for a novice to be able to learn. However, while we would agree that expertise develops with time, it was not the aim of this study to produce experts rather it was to determine if it is possible to enhance interpretative skills and confidence in x-ray interpretation.

This study did incorporate a subjective analysis of confidence level changes over time, therefore the association between subjective confidence level may not reflect the objective performance. Additionally, the cases provided in the pre-test and post-test were identical; hence there is likely to be a ‘priming’ effect of the test/retest.

Furthermore, not all students in this study completed the assessments at each time point. However, there were no significant differences in intervention scores for those who participated in all intervention assessments and those who did not.

Finally, while the survey instruments and e-learning assessment module are valid i.e. designed by experts, mapped to educational content, had clear instructions, a simple assessment rubric and demonstrated improvement with training, scoring was undertaken by one single assessor ensuring consistency but also limiting more detailed reliability analysis. Further testing must therefore be carried out on the assessment instrument to ensure appropriate reliability if to be used in the future.

## Conclusion

This study highlights the importance of early exposure to properly integrated radiology teaching and interpretation in medical school curricula. Not only can increasing students’ radiology exposure in their undergraduate training improve their diagnostic abilities, but it can also increase their confidence.

The positive feedback received from students, the improvement in interpretation skills and the increased confidence levels demonstrated by students suggest that more e-learning radiology modules could be greatly beneficial in undergraduate medical school curricula. Further studies are needed to fully explore the perceptions of medical students and faculty with respect to radiology teaching methods and to assess the long-term retention of radiological interpretation skills following the use of an e-learning x-ray interpretation module.

## Additional files

Additional file 1: Appendix 1. CXR Interpretation: The Heart. This appendix depicts the assessment sheet that students were required to complete for each of the cases related to the module 'CXR: Interpretation of the Heart'. (DOCX 17 kb)

Additional file 2: Appendix 2. CXR Interpretation Questionnaire. This appendix depicts the questionnaire that was made available for participants to provide feedback regarding their confidence in interpreting chest x-ray images with particular focus on interpretation of the heart. (DOCX 17 kb)

## Abbreviations

ANOVA: Analysis of variance
BM BS: Bachelor of Medicine, Bachelor of Surgery
CHF: Congestive Heart Failure
GEMS: Graduate Entry Medical School
NFQ: National Framework Qualification
PA: Postero-anterior
PBL: Problem Based Learning
SD: Standard Deviation
UL: University of Limerick
