# Supplementary material for: E-learning for chest x-ray interpretation improves medical student skills and confidence levels
Source: BMC Med Educ. 2018 Nov 12;18:256. doi: 10.1186/s12909-018-1364-2 (PMC6233516; doi:10.1186/s12909-018-1364-2)
Supplement: Supplementary file 1 — Appendix 1. CXR Interpretation: The Heart. This appendix depicts the assessment sheet that students were required to complete for each of the cases related to the module 'CXR: Interpretation of the Heart'. (DOCX 17 kb) [file 12909_2018_1364_MOESM1_ESM.docx]

**CXR Interpretation: The Heart**

Student number __________________________________

**The following assessment is not summative and will not affect your final grade. It is solely to evaluate any prior chest x-ray (CXR) knowledge and determine if it would be beneficial to incorporate more e- tutorials on CXR interpretation relating to PBL cases into the GEMS Programme. Your student number is required so that we can match your responses pre and post accessing the e-tutorial.**

| CASE 1 | | |
| --- | --- | --- |
| Grayscale | - Too white? - Too black? - Normal? | ***Special Case CHF:***   - Enlargement of pulmonary veins? - Kerley B lines? - Hilar Haze? - Bronchial Cuffing? - Alveolar Oedema? |
| Size | - Too large? - Normal? |  |
| Shape | - Distorted shape? - Normal? |  |
| Position | - Shifted from left of centre? - Normal? |  |
| POSSIBLE DIAGNOSIS: | | |

| CASE 2 | | |
| --- | --- | --- |
| Grayscale | - Too white? - Too black? - Normal? | ***Special Case CHF:***   - Enlargement of pulmonary veins? - Kerley B lines? - Hilar Haze? - Bronchial Cuffing? - Alveolar Oedema? |
| Size | - Too large? - Normal? |  |
| Shape | - Distorted shape? - Normal? |  |
| Position | - Shifted from left of centre? - Normal? |  |
| POSSIBLE DIAGNOSIS: | | |

| CASE 3 | | |
| --- | --- | --- |
| Grayscale | - Too white? - Too black? - Normal? | ***Special Case CHF:***   - Enlargement of pulmonary veins? - Kerley B lines? - Hilar Haze? - Bronchial Cuffing? - Alveolar Oedema? |
| Size | - Too large? - Normal? |  |
| Shape | - Distorted shape? - Normal? |  |
| Position | - Shifted from left of centre? - Normal? |  |
| POSSIBLE DIAGNOSIS: | | |

| CASE 4 | | |
| --- | --- | --- |
| Grayscale | - Too white? - Too black? - Normal? | ***Special Case CHF:***   - Enlargement of pulmonary veins? - Kerley B lines? - Hilar Haze? - Bronchial Cuffing? - Alveolar Oedema? |
| Size | - Too large? - Normal? |  |
| Shape | - Distorted shape? - Normal? |  |
| Position | - Shifted from left of centre? - Normal? |  |
| POSSIBLOE DIAGNOSIS: | | |

| CASE 5 | | |
| --- | --- | --- |
| Grayscale | - Too white? - Too black? - Normal? | ***Special Case CHF:***   - Enlargement of pulmonary veins? - Kerley B lines? - Hilar Haze? - Bronchial Cuffing? - Alveolar Oedema? |
| Size | - Too large? - Normal? |  |
| Shape | - Distorted shape? - Normal? |  |
| Position | - Shifted from left of centre? - Normal? |  |
| POSSIBLE DIAGNOSIS: | | |
