# Supplementary material for: E-learning for chest x-ray interpretation improves medical student skills and confidence levels
Source: BMC Med Educ. 2018 Nov 12;18:256. doi: 10.1186/s12909-018-1364-2 (PMC6233516; doi:10.1186/s12909-018-1364-2)
Supplement: Supplementary file 2 — Appendix 2. CXR Interpretation Questionnaire. This appendix depicts the questionnaire that was made available for participants to provide feedback regarding their confidence in interpreting chest x-ray images with particular focus on interpretation of the heart. (DOCX 17 kb) [file 12909_2018_1364_MOESM2_ESM.docx]

**CXR Interpretation Questionnaire**

Please rate your level of confidence in your ability to complete the following tasks on a scale 1(not at all confident) to 5(extremely confident).

|  | Not at all confident |  |  |  | Extremely confident |
| --- | --- | --- | --- | --- | --- |
| Determining if the CXR is normal or abnormal | 1 | 2 | 3 | 4 | 5 |
| Determining if the heart is too white, too black or of normal grayscale | 1 | 2 | 3 | 4 | 5 |
| Determining if the heart is too large or of normal size | 1 | 2 | 3 | 4 | 5 |
| Determining if the heart is of normal or distorted shape | 1 | 2 | 3 | 4 | 5 |
| Determining if the heart is in a normal anatomical position or if it has shifted | 1 | 2 | 3 | 4 | 5 |
| Giving a differential diagnosis based on the image findings | 1 | 2 | 3 | 4 | 5 |
| Overall interpretation of the heart on a CXR | 1 | 2 | 3 | 4 | 5 |

Would more tutorials on CXR interpretation relating to PBL cases benefit your learning? **YES NO**

Would you recommend this tutorial to a friend? **YES NO**

Is there anything additional that could be incorporated into the tutorials to benefit your learning of CXR interpretation? __________________________________________________________________________________________________________________________________________________________________________
